# Lipidomic Analysis of TRPC1 Ca^2+^-Permeable Channel-Knock Out Mouse Demonstrates a Vital Role in Placental Tissue Sphingolipid and Triacylglycerol Homeostasis Under Maternal High-Fat Diet

**DOI:** 10.3389/fendo.2022.854269

**Published:** 2022-03-10

**Authors:** Michael R. Bukowski, Brij B. Singh, James N. Roemmich, Kate J. Claycombe-Larson

**Affiliations:** ^1^ USDA-ARS Grand Forks Human Nutrition Research Center, Grand Forks, ND, United States; ^2^ School of Dentistry, UT Health Science Center San Antonio, San Antonio, TX, United States

**Keywords:** infusion lipidomics, placental lipidome, TRPC1, sphingolipid metabolism, triacylglycerol

## Abstract

The transient receptor potential canonical channel 1 (TRPC1) is a ubiquitous Ca^2+^-permeable integral membrane protein present in most tissues, including adipose and placenta, and functionally regulates energetic homeostasis. We demonstrated that elimination of TRPC1 in a mouse model increased body adiposity and limited adipose accumulation under a high fat diet (HFD) even under conditions of exercise. Additionally, intracellular Ca^2+^ regulates membrane lipid content *via* the activation of the protein kinase C pathway, which may impact placental membrane lipid content and structure. Based upon this we investigated the effect of HFD and TRPC1 elimination on neutral lipids (triacylglycerol and cholesteryl ester), membrane lipids (phosphatidylcholine and phosphatidylethanolamine), and other multifunctional lipid species (unesterified cholesterol, sphingomyelins, ceramides). The concentration of unesterified cholesterol and sphingomyelin increased with gestational age (E12.5 to E 18.5.) indicating possible increases in plasma membrane fluidity. Diet-dependent increases ceramide concentration at E12.5 suggest a pro-inflammatory role for HFD in early gestation. TRPC1-dependent decreases in cholesterol ester concentration with concomitant increases in long-chain polyunsaturated fatty acid -containing triacylglycerols indicate a disruption of neutral lipid homeostasis that may be tied to Ca^2+^ regulation. These results align with changes in lipid content observed in studies of preeclamptic human placenta.

## Introduction

High fat diet (HFD)-fed mothers and fathers are obese, hyperglycemic, and hyperlipidemic (1). A parental HFD also contributes to gestational programing of offspring obesity (2–5). We have demonstrated that TRPC1 increases total body adiposity in mice by decreasing the efficacy of exercise to limit adipose accumulation under a HFD (6). Placental lipid regulation is vital to the development of a healthy placenta and fetus. Obesity and a HFD are associated with increased risks of preeclampsia, gestational diabetes, placental inflammation, and fetal macrosomia (7–9). Even during healthy pregnancies, placentae from obese women contain 20% more esterified lipid than placental samples from non-obese women, which impairs placental function (10). Investigating mechanistic relationships among adiposity, placental function, and putative placental tissue lipid content-associated placental dysfunction are important for understanding how HFD-induced obesity may impact fetal development mediated *via* placental dysfunction.

We have demonstrated that adipose proliferation is reduced in mice by knocking-out transient receptor potential canonical channel 1 (TRPC1) (11). TRPC1, a ubiquitous member of the transient receptor potential superfamily, is an integral membrane protein that regulates Ca^2+^ ion flux across the membrane (12). We have demonstrated that TRPC1 -/- animals have reduced adipocyte differentiation, reduced markers for autophagy, and increased expression of apoptosis markers, suggesting a change in neutral lipid storage and the sphingomyelin-ceramide pathway regulating nutrient transport, cell proliferation, and apoptosis (13, 14). We also demonstrated lower adipose mass in HFD fed TRPC1 -/- mice when compared to HFD fed WT mice (11, 15). When provided access to exercise, HFD fed TRPC1 -/- mice experienced greater loss of adipose by mass and reduced insulin resistance compared to WT animals under the same conditions, indicating TRPC1 plays a role in systematic energy homeostasis, particularly neutral lipid metabolism.

The hydrolysis of membrane-bound sphingomyelin (SM) to generate the secondary messenger ceramide (Cer) is a highly conserved signaling pathway that responds to cellular stress and regulates apoptosis (13). We hypothesized that placentae from TRPC1 -/- dams have increased Cer concentration when compared to control animals due to the metabolic changes we have previously observed in this mouse model (11, 15). If this is the result of increased SM hydrolysis, then there should be a concomitant decrease in SM concentration. Using a HFD high in saturated fat, there should be an additive increase in Cer concentration (16). Additionally, we aimed to determine whether the absence of TRPC1 gene in placentae altered the composition of neutral lipid storage. To test this hypothesis, we developed a comprehensive infusion lipidomic workflow which measured Cer, SM, phosphatidylcholines (PC), phosphatidylethanolamines (PE), triacylglycerols (TAG), cholesterol esters (CE), and unesterified cholesterol (FC).

## Experimental

### Animal Protocol Design and Approval

Two-month-old female B6129SF2/J mice (Envigo, Indianapolis, IN) were fed diets containing either 16% (normal-fat, NF) or 45% fat (high-fat, HF) for 12 weeks (**Table S1**). Following 12 weeks of diet intervention, dams were bred with normal chow fed males and pregnancy identified by the presence of a vaginal plug. Midday identification of the vaginal plug was considered embryonic day 0.5 (E0.5). Dams were maintained on their respective diets throughout mating and pregnancy. Dam euthanasia was by CO_2_ inhalation according to the animal use and care protocol approved by the USDA Agricultural Research Service, Grand Forks Human Nutrition Research Center Animal Care and Use Committee. Fetuses and placentae were harvested in mid-gestation (E12.5-E13.5) or late gestation (E18.5-E19.5), weighed, measured, and then immediately frozen in liquid nitrogen.

### Placental and Fetal Tissue Weight Measurements

The uterine horn was dissected from the dam and cut between each implantation site separating each amniotic sac containing individual fetus. Removed placenta and fetus were blotted dry before removal of umbilical cords. Weights were recorded and the placenta length and width were measured with the maternal convex side up using a digital caliper (Marathon Watch Company LTD., Richmond Hill, ON Canada). Samples were then flash frozen in liquid nitrogen and stored at -80°C.

### Placental Lipid Analysis

HPLC-grade isopropanol, chloroform, butylated hydroxytoluene, and hexane were purchased from Sigma Aldrich (St. Louis, MO, USA) and used as received. HPLC-grade methanol was purchased from Honeywell (Muskegon, MI, USA). Silicic acid (200-325 mesh) was ordered from Clarkson Chromatography Products Inc. (South Williamsport, PA, USA). Internal standards for TAG and CE analysis were purchased from NuChek Prep Inc. (Elysian, MN, USA), phospholipid and sphingolipid standards and LIPIDMAPS standards were purchased from Avanti Polar Lipids (Alabaster, AL, USA) (**Table S2**).

Briefly, frozen placental samples were homogenized in aqueous buffer. Neutral lipid internal standards were added, and lipids were extracted in 3:2 hexane: isopropanol (50 µmol/L BHT). Extracts were isolated and dried under, then redissolved in 1 mL chloroform. The solution was divided in half, with 500 µL retained for phospholipid analysis (below). Phospholipids were removed from the neutral lipid fraction by dispersive SPE with silicic acid, and100 µL of the supernatant was combined with 100 µL of methanol (20 mM ammonium acetate). This sample (Solution 1) was analyzed for TAG and CE content as described below. A 500 µL aliquot of the SPE supernatant was used for the assay of FC following the method of Liebisch et al. (17).

Analysis of polar lipids was performed using the method adapted from Sundaram et al. with modifications made to allow for automation (18).

### Data Collection and Analysis

Data were collected on an AB Sciex 5500 QTRAP hybrid mass spectrometer equipped with a Turbo V electrospray ion source and SelexION ion mobility device (AB Sciex, Framingham, MA, USA). Samples were infused using a Shimadzu Prominence UPLC system (Shimadzu Scientific Instruments, Columbia, MD, USA) equipped with an LC20XR autosampler (50 µL stainless steel sample loop) and two solvent delivery units following a configuration modified from Bukowski and Picklo as detailed in the **Supplemental Information** (19).

Multiple instrument modes were used based upon lipid class and preparation (**Table S2**). TAG were characterized and quantitated by brutto structure as ammoniated cations, [TAG + NH_4_]^+^ in enhanced mass spectrum (EMS) mode as previously published (19, 20). Cholesterol esters (CE) were assayed by neutral loss scan for 20 fatty acid neutral losses with confirmatory detection of the cholesterol head group by product ion scan for mass-to-charge ratio (m/z) = 369, as previously published (**Table S3**) (20, 21). Acylated samples for FC determination were analyzed using multiple reaction monitoring for the acylated d7-cholesterol species (*m/z* = 453.4
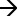
376.3) and the endogenous acylated cholesterol (*m/z* = 446.4
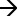
369.3) (17). Ceramide species (Cer) were detected using the product ion m/z 264, selective to the sphingosine backbone, and quantified following the method of Picklo *et al.* (22). Phosphatidylethanolamine, and phosphatidylcholine species were quantified as brutto species using our previously published methods (20, 23). Monitoring the neutral loss of 141 Da allowed for the selective measurement of PE species, while monitoring the product ion m/z = 184 was selective for PC and SM species.

SM species were isolated for characterization and quantitation using the Selexion ion mobility device (24).

Mass spectra for all lipid class were examined manually to confirm brutto structure assignment. Isotopic and ionization correction factors were determined as previously described and quantitation of target species was performed using LipidView software (AB Sciex, Framingham, MA, USA) (19, 20, 22, 25). Values were normalized to tissue wet weight. TAG 54:0 and TAG 54:1 were excluded from analysis due to isobaric overlap with a silicone oligomer contaminant which was extracted from the septa and could not be reliably removed by subtraction.

### Statistical Analysis

Statistical analyses were performed using MetaboAnalyst 5.0 (26). After range scaling, data were analyzed by one-way ANOVA with an alpha of P<0.05, and a 0.05 false discovery rate. Tukey’s HSD was employed to as a *post-hoc* test for significant interactions between groups.

## Results

### Types and Amount of Placental Lipid Determination

Using the combined infusion mass spectrometric methods 190 major lipid species including sphingolipids and phospholipids were identified in the placental samples. In the neutral lipid fraction 34 cholesterol esters, 50 TAG and FC were quantitated (additional details provided in **Tables S4, S5**). In the polar lipid fraction, brutto structures included 49 PC, 36 PE, eight SM, eight Cer, and four HexCer species (**Tables S6–S8**, respectively). A summary of the relationships established by univariate analysis is presented in **Table 1**.

**Table 1:** Summary of lipid classes and relationships observed by 1-way ANOVA (p < 0.05) with.

Relationship		Lipid Class								
		FC	CE	TAG	PC	PE	SM	CER	HexCer	Total
WT -HF-M vs. WT -HF-L	Gestation (*)	1	1	3	1	1	7	3	0	17
WT -NF-M vs. WT -NF-L		1	10	4	1	1	7	0	1	25
TRPC1 -/- -HF-M vs. TRPC1 -/- -HF-L		1	14	2	14	10	7	3	0	51
TRPC1 -/- -NF-M vs. TRPC1 -/- -NF-L		1	0	2	2	0	6	7	1	19
TRPC1 -/- -NF-L vs. TRPC1 -/- -HF-L	Diet (**)	0	15	0	0	0	7	0	0	22
TRPC1 -/- -NF-M vs. TRPC1 -/- -HF-M		0	0	4	10	2	3	7	0	26
WT -NF-L vs. WT -HF-L		0	3	3	2	0	3	5	0	16
WT -NF-M vs. WT -HF-M		0	24	1	1	0	1	8	0	35
WT -HF-L vs. TRPC1 -/- -HF-L	Genotype (‡)	0	1	15	0	0	1	0	0	17
WT -HF-M vs. TRPC1 -/- -HF-M		0	10	0	5	4	1	1	0	21
WT -NF-L vs. TRPC1 -/- -NF-L		0	6	9	1	0	3	4	0	23
WT -NF-M vs. TRPC1 -/- -NF-M		0	0	1	0	0	0	0	0	1

Symbols indicate differences with p<0.05 base upon one-way ANOVA with Tukey’s HRD post hoc test and application of a 0.05 false discovery rate for gestational age (*), diet (**), and genotype (‡). P-values are available in **Supplemental Materials**. WT, wild-type; TRPC1 -/-, TRPC1 knock-out; NF, normal fat diet; HF, high-fat diet, M, mid-gestation; L, late gestation.

**Figure 1:**
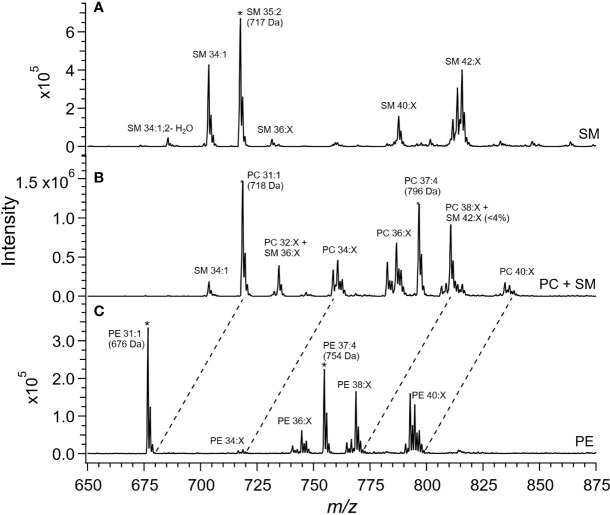
Mass spectra of placental extracts from NF-WT mouse. Selective scans for **(A)** sphingomyelin (SM) using the product ion *m/z* 184 with differential ion mobility, **(B)** phosphatidylcholine and sphingomyelin using product ion *m/z* 184, and **(C)** phosphatidylethanolamine using the neutral loss of 141 Da. Internal standards are indicated with *. Dashed lines indicate glycerophospholipid groups with common acyl carbon number.

Cer and other glycosphingolipids (cerebroside) species were identified based upon the m/z = 264 product ion which was selective for the sphingosine base, d18:1, allowing for identification of the fatty acid moiety based upon the precursor ion scan for the Cer species. As shown in **Figure 1**, PC and SM species were both detected in the precursor ion scan. The monoisotopic peaks used for quantitation of SM and PC species were offset one Dalton and presented overlap as in the case of the grouping of peaks from m/z 806-816, in which species PC 38:4, PC 38:3, PC 38:2, and PC 38:1 overlapped with SM 42:3, SM 42:2, and SM 42:1. Removal of PC the contribution using the Selexion ion mobility interface was necessary to accurately quantitate to SM, which represented approximately 4% of the signal. A difference in acyl carbon distribution between PC and PE species was evident (**Figure 1**).

When compared, PE and PC species with equivalent acyl carbon numbers in placental membrane phospholipids demonstrated an increase in concentration of LC-PUFA moieties in PE (**Figure 1**, dashed lines). The majority of PC species had 34, and 36 acyl carbons with between 0 and 3 double bonds, corresponding to structures containing 16- and 18-carbon fatty acids such as palmitic, palmitoleic, stearic, oleic, and linoleic acids while the dominant PE species had 40 acyl carbons and between 4 to 7 double bonds and were composed largely of PUFA species as detailed in the **Supplemental Information** (**Table S9**) (25).

### Lipid Content and Species Distribution Was Affected by Gestational Age

**Figure 2:**
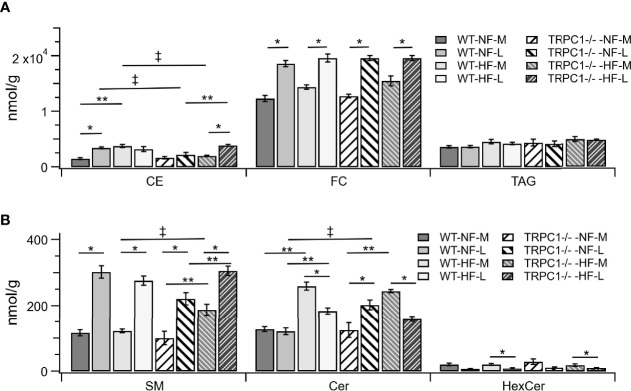
Lipid class differences between groups for **(A)** cholesterol esters (CE), unesterified cholesterol (FC), triacylglycerols (TAG). Sphingolipids are shown in **(B)**, sphingomyelin (SM), ceramide (Cer), and hexosylceramides (HexCer). Data are shown as mean ± sem (n = 8, except for TRPC1 -/- -HF-M where n = 4). Symbols indicate differences with p<0.05 base upon one-way ANOVA with Tukey’s HRD *post hoc* test and application of a 0.05 false discovery rate for gestational age (*), diet (**), and genotype (‡). WT, wild-type; TRPC1 -/-, TRPC1 knock-out; NF, normal fat diet; HF, high-fat diet; M, mid-gestation; L, late gestation.

**Figure 3:**
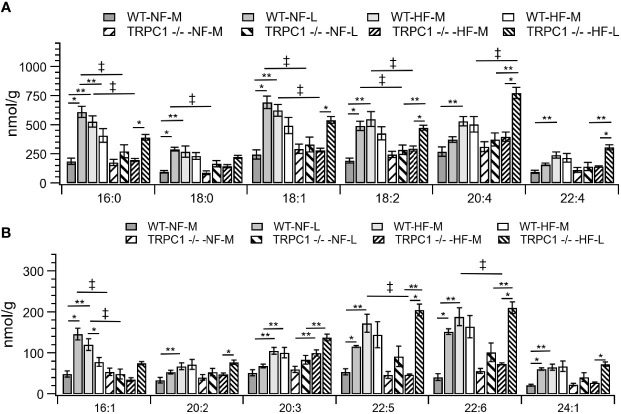
Placental CE concentration by species for CE C:N. C = acyl carbon number and N = acyl desaturation level. Species are grouped with concentration decreasing from **A** to **B** to demonstrate the range of concentrations. Data are shown as mean ± sem (n = 16, except for HF-TRPC1 where n = 12). Symbols indicate differences with p<0.05 base upon one-way ANOVA with Tukey’s HRD *post hoc* test and application of a 0.05 false discovery rate for gestational age (*), diet (**), and genotype (‡). P-values are available in **Supplemental Materials**. WT, wild-type; TRPC1 -/-, TRPC1 knock-out; NF, normal fat diet; HF, high-fat diet, M, mid-gestation; L, late gestation.

The gestational age of the placenta affected the neutral lipid and sphingolipid content. For neutral lipid species the concentration of FC (**Figure 2A**) increased from mid-gestation to late-gestation irrespective of diet or genotype. While animals on HFD appeared to have greater FC at mid gestation, biological variability rendered this difference non-significant and placental FC concentrations at late gestation were indistinguishable. A gestational age-dependent increase in total CE concentration was observed for WT-NF and TRPC1 -/- -HF animals. For the WT-NF group the increase was due primarily to increases in major species such as CE 16:0, CE 18:0, CE 18:1, CE 18: 2, (**Figure 3A**), though gains in CE 16:1, CE 20:2, CE 22:5, CE 22:6 and CE 24:1 also contributed to the elevated concentration. The major contributor to the increase in CE concentration for TRPC1 -/- -HF animals was CE 20:4, which increased 1.9-fold from mid to late gestation. Gestation-dependent increases in CE 22:5 (4.3-fold, **Figure 3B**) and CE 22:6 (2.9-fold, **Figure 3B**) and CE 22:4 (2.6-fold, **Figure 3A**) were also observed. These trends were not observed for either the TRPC1 -/- -NF or WT-HF animals, though the latter did exhibit a decrease CE 16:1 concentration (**Figure 3B**).

**Figure 4:**
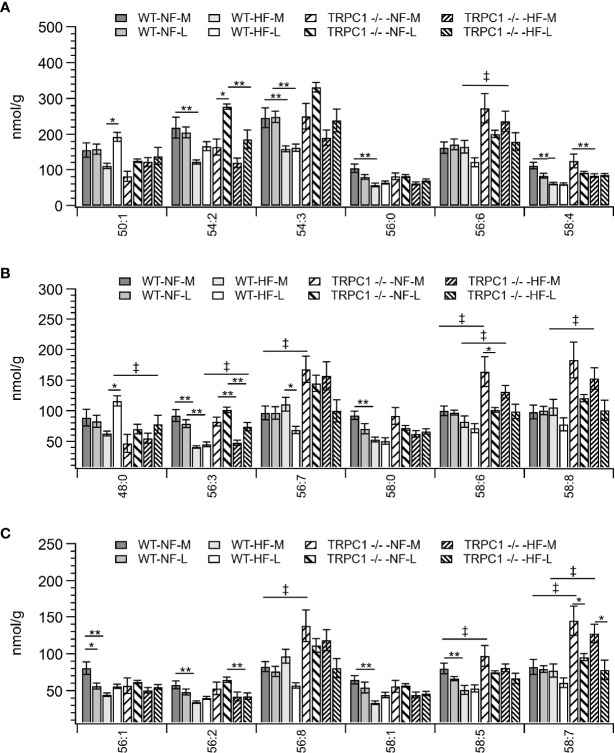
Placental TAG concentration by species for TAG C:N. C = acyl carbon number and N = acyl desaturation level. Species are grouped with diminishing concentrations from **A**–**C** to illustrate the range of concentrations. Data are shown as mean ± sem (n = 16, except for HF-TRPC1 where n = 12). Symbols indicate differences with p<0.05 base upon one-way ANOVA with Tukey’s HRD *post hoc* test and application of a 0.05 false discovery rate for gestational age (*), diet (**), and genotype (‡). P-values are available in **Supplemental Materials**. WT, wild-type; TRPC1 -/-, TRPC1 knock-out; NF, normal fat diet; HF, high-fat diet; M, mid-gestation; L, late gestation.

Total placental TAG concentration was not affected by gestational age; however, individual TAG species underwent gestation-dependent changes. Tissue from WT-HF dams demonstrated increased concentrations of TAG 50:1 and TAG 48:0 with a concomitant decrease in TAG 56:7 (**Figures 4A, B**). For WT animals, irrespective of diet, the most concentrated TAG species, (TAG 54:2, TAG 54:3, TAG 56:6, TAG 56:8, TAG 58:6, TAG 58:8) were stable over the observed gestational time scale, however TRPC1-knockout animals experienced decreases in TAG 58:6 and TAG 58:6 (**Figures 4B, C**), though the concentrations of these species were higher at the early gestational time point, as will be discussed below.

**Figure 5:**
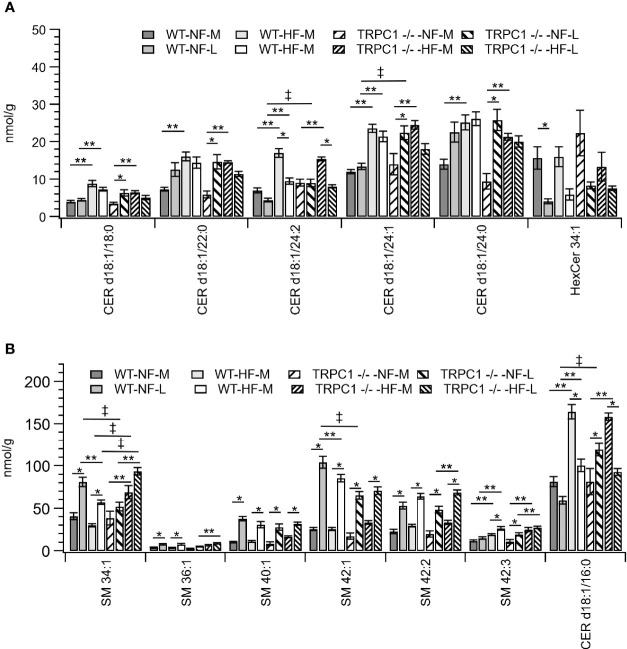
Placental sphingolipid concentration by species. **(A)** Cer, and **(B)** SM species. Data are shown as mean ± sem (n = 8, except for HF-TRPC1-M where n = 4). Symbols indicate differences with p<0.05 base upon one-way ANOVA with Tukey’s HRD *post hoc* test and application of a 0.05 false discovery rate for gestational age (*), diet (**), and genotype (‡). P-values are available in **Supplemental Materials**. WT, wild-type; TRPC1 -/-, TRPC1 knock-out; NF, normal fat diet; HF, high-fat diet; M, mid-gestation; L, late gestation.

Gestational age affected the concentration of sphingolipids in placentae. In all cases the SM concentration increased with gestational age (**Figure 2B**). The major contributors to the net increase in SM concentration were SM 42:1, SM 42:2, and SM 34:1 (**Figure 5B**). Total Cer concentrations were unchanged for WT-NF samples but increased with gestational age for TRPC1 -/- -NF (**Figure 2B**). Cer d18:1/16:0, Cer d18:1/22:0, Cer d18:1/24:0, and Cer d18:1/24:1 were the major contributors to this increase (**Figure 5A**) Contrastingly, the WT-HF and TRPC1 -/- -HF arms exhibited decreased Cer concentration with increasing gestational age, as well as a decrease in total HexCer concentration.

Total concentration PE species were not affected by gestational age in aggregate (**Figure S1**); however, PE 40:4 decreased in concentration for nearly all groups with increasing gestational age (**Figure S2B**). Only placentae from TRPC1 -/- -HF dams demonstrated a loss in PC concentration (**Figure S1**) but the concentration major contributors to that change were PC 36:2, PC 36:3, PC 38:3, and PC 34:2 (**Figures S3A, B**).

### Lipidomic Responses to HFD Indicate Difference in Neutral Lipid Storage Between WT and TRPC1 -/- Placentae

Cholesterol ester concentrations at the earlier gestational time point were greater (p<0.05) in placentae from HFD fed dams irrespective of phenotype (**Figure 2A**). Species-level univariate analysis revealed the diet-induced increase in cholesterol ester concentration was independent of fatty acid identity, with increases in concentration for nearly all cholesterol ester species (**Figure 3**). The exceptions to this trend were CE 16:0 and CE 16:1, which demonstrated decreases from WT-NF -L to WT-HF-L. Tissue from TRPC1 -/- -HF-L dams demonstrated increases in CE 18:2, CE 20:4, CE 22:4, CE 20:3 CE 22:5, and CE 22:6 relative to TRPC1 -/- -NF-L though, with the exception of CE 20:4, the concentrations at this time point were not significantly different from WT-HF-L samples.

Differences in genotypic response to HFD were observed between the WT-HF-M and TRPC1 -/- -HF-M arms (**Figures 3A, B**). At the earlier time point the SFA-containing species CE 16:0 and CE 18:0 were only 37% and 53% of the WT-HF-L arm, respectively. This trend held for MUFA-containing CE (CE 18:1 and CE 16:1; 53% and 30% of WT-HF-L, respectively), as well as PUFA-containing CE species such as CE 18:2, CE 20:5, CE 22:5, and CE 22:6 (54%, 54%, 28%, and 39% of WT-HF-L, respectively). These differences were not present at the later time point for the HFD arms. These data, along with the observed increase in CE 20:4 noted earlier suggests a compensatory mechanism for PUFA CE accumulation.

For samples from WT animals consumption of HFD led to a decrease in TAG storage for TAG 54:2, 54:3, 54:7, 54:9, 56:0, 56:1, 56:2, 56:3, 56:9, 56:10, 58:0, 58:1,58:2, 58:4, 58:5, 58:10, 58:11, 60:11, and 60:12 (**Figures 4A–C** and **Figure S2**). TAG species which displayed genotypic responses followed the pattern of presenting elevated TAG levels for the TRPC1 -/- placentae relative to the WT at the earlier gestational time point. These species included TAG 56:3, 56:6, 56:7, 56:8, 58:5, 58:6, 58:7, 58:8, 58:10, 60:11, and 60:12 (**Figures 4A–C** and **Figure S2**). TAG 48:0 presented as an outlier to this trend presenting greater in WT-HF-L animals that the corresponding TRPC1 -/- arm (**Figure 4B**).

### Sphingolipid Concentrations in Placental Tissue Were Altered by Gestation, Diet, and Genome

As discussed above, the general trend observed for SM species was an increase in concentration with gestational age irrespective of diet or genotype. For SM 34:1 a genotype-dependent response was observed. At the later gestation time point the concentration of SM 34:1 for TRPC1 -/- -NF-L was 64% of that observed for the corresponding WT-NF-L arm, however TRPC1 -/- -HF-M and TRPC1 -/- -HF-L had SM 34:1 concentrations that were 230% and 160% greater than in the corresponding WT groups (**Figure 5B**).

In addition to the gestational trends indicated above, the concentration of Cer species tended to be higher in placentae from WT-HF animals. For TRPC1 -/- -NF-L subjects the concentrations of Cer d18:1/16:0, Cer d18:1/24:1, and d18:1/24:1 increased with gestational age and were greater than corresponding WT-NF-L subjects (**Figure 5B**).

## Discussion

This is the first analysis of mouse placentae at two gestational time points that demonstrates the effect of HFD on lipidomic profiles. Our first measurement at E12.5 corresponds to the appearance of the first definitive placenta, while the second measurement point near E18.5 represents a fully functioning organ nearing the end of its lifespan, preparing for parturition (27, 28). The visibly greater concentration of FC between the two diet arms at the early time point was not present by E18.5. Perhaps a nutritional sensitivity for FC early in pregnancy diminishes as the placenta develops and regulates cholesterol homeostasis and cholesterol efflux to the fetus. This could in part explain why, in a rodent model, cholesterol supplementation of the maternal diet impacts the serum lipid profile (HDL, LDL, TG, total cholesterol) without altering the fetal lipid profile (29). and the decrease in serum lipid concentrations post parturition in humans (30). Increased FC may also reflect greater membrane fluidity during placental development (31). Future work evaluating the impact of HFD on the transcription of enzymes involved in the biosynthesis and transport of cholesterol such as DHCR-7, Abca1, Abcg1 and Sr-b1 would shed light on the underlying mechanisms of this observation.

Our CE results also indicate a role for the placenta in fetal lipid homeostasis under HF conditions. While the placentae from WT-NF dams increased in CE concentration with gestational age, under WT-HF conditions the CE concentration decreased with gestational age, particularly for CE 16:1. Palmitoleic acid is a marker for *de novo* lipogenesis (DNL) (32), and a HFD suppresses DNL (33), specifically for lard-based diets such as the one used in this study (34). Conversely, HFD does not suppress TAG synthesis, thus TAG composition reflects re-esterification of diet-derived fatty acids (18, 33). As expected, we observed no diet-dependence on overall TAG concentration, however it was not possible to evaluate the second hypothesis as a lard-based diet is reflective of the endogenous fatty acids typical to most mammals. Additional work should be done to evaluate whether the down-regulation of DNL is of placental or hepatic origin.

The greater concentration of SM in the gestationally more developed placentae is likely a consequence of increased formation of lipid raft domains to process signaling and vesicle transport across the plasma membrane (35). Indeed, SM is critical for the function of TRP cation channels (36). In the fully developed placentae (D18.5) from TRPC1 -/- -NF dams the concentration of major SM species (SM 34:1 and SM 42:1) were lower than for WT-NF, due to a reduction in the presence of lipid raft domains coincident with the elimination of a TRP cation channel. SM species also act as substrate for initiation of sphingolipid signaling (35). Thus, the greater concentrations of SM 34:1 in TRPC1 -/- -HF placentae relative to WT-HF at both developmental periods suggests perturbation of sphingolipid homeostasis independent of gestational status. This is an important finding because Del Gaudio and colleagues noted accumulation of SM 34:1, SM 36:1 and SM 42:1 to the endothelium of feto-placental blood vessels from preeclamptic placentae, indicating a link between SM-accumulation and placental vascular development (37).

SM catabolism is the first step in a sphingolipid signaling cascade initiated by Toll-like receptor 4 (TLR4), a well-known pro-inflammatory mediator of innate immunity (16). The greater Cer concentration in HF-WT dams at both gestational periods (**Figure 2B**) mirrored the results observed by Holland et al. (16) in multiple tissues in response to an infusion of high-SFA lard oil. This is also in agreement with the pro-inflammatory role of diets high in SFA (38–40). The subsequent decrease in Cer concentration with increasing gestational age for both WT-HF and TRPC1 -/- -HF animals suggests a conserved role for Cer signaling in energetic homeostasis (14, 41). The increase in Cer species for the TRPC1 -/- -NF arm of the study may indicate in increased inflammatory load for these animals, though further characterization of inflammatory markers is needed to confirm this possibility.

TRPC1 mRNA and protein are expressed in human placentae (42) and upregulation of TRPC1 protein and subsequent increase in Ca^2+^ influx into the placental tissue may be a crucial step for decidualization (43). However, it is not yet known whether the absence of TRPC1 affects placental function *via* changes in placental lipid content, especially under maternal HFD. Data presented in our study showed placental lipid composition is modifiable due to absence of TRPC1 expression under maternal HFD, particularly at E12.5 of gestation. The decrease in cholesterol ester concentration for TRPC1 -/- animals (**Figures 3A, B**) with no concomitant genotype-dependent change in FC suggests either a decrease in CE formation or an increase in CE hydrolysis. Further investigation is needed to determine the role of enzymes that generate CE (lecithin cholesterol acyltransferase, sterol O-acyltransferase), or hydrolyze CE (cholesterol ester hydrolase). The increase in long-chain CE species for the TRPC1 -/- -HF-L arm indicates a possible compensatory mechanism for the formation of long-chain PUFA species.

For TRPC1 -/- animals we observed increased concentrations for TAG species with 56 or more acyl carbons and greater than 4 points of desaturation. In previous work it was observed that species in this regime contain long-chain PUFAs (18, 20). Diets were not supplemented with additional PUFA, which suggests this increase results from increased fatty acid elongation and TAG synthesis (33). We have observed PPARγ-dependent down regulation of oxidative metabolism in brown adipose from TRPC1-deficient mice (44) and reduced autophagy in adipose tissue (15), thus this may be an adaptation to sequester fatty acids as TAG species in lipid droplets to forestall the formation of a lipotoxic environment. Transcriptomic analysis for expression of sterol-regulatory element binding protein 1 (SREBP-1), sterol-CoA desaturase (SCD-1), diacylglycerol acyltransferases (DGAT1 and DGAT2), and ELOVL elongases would allow for the testing of this hypothesis. Unesterified fatty acids could also be assayed by fatty acid methyl ester analysis, though it would necessitate a larger sample collection that was available.

Our observation of TAG accumulation is congruent with observations from a study of functional complications in human placentae. Using infusion lipidomic methods like those employed in our study Brown et al. analyzed human placental biopsies from heathy pregnancies and pregnancies with complications due to preeclampsia or intrauterine growth restriction (45). Preeclampsia and intrauterine growth restriction were associated with greater TAG content, specifically in PUFA-containing species.

Lipidomic analysis presents an important window into energetic homeostasis, placental development, and inflammatory status, but complimentary techniques are needed to support some of the arguments above. The neutral lipid products quantified above implicate several enzymatic pathways for CE, TAG and fatty acid synthesis which could be addressed using transcriptomic analysis (46). Placental inflammation and vascular development could be assessed as we have in prior work (47). Limitations in sample availability preclude following up on these lines of inquiry for this study.

## Conclusions

We performed an infusion based lipidomic analysis to determine the effects of gestational age, diet and elimination of the TRPC1 Ca^2+^ transport mechanism on the placental lipidome. Increasing gestational age resulted in increased unesterified cholesterol and sphingomyelin that may reflect increased plasma membrane fluidity and cross-membrane signaling. Changes in cholesterol ester and TAG content indicate the disruption of the TRPC1 Ca^2+^ may promote increased TAG storage.

## Data Availability Statement

The original contributions presented in the study are included in the article/**Supplementary Material**. Further inquiries can be directed to the corresponding authors.

## Ethics Statement

The animal study was reviewed and approved by USDA Agricultural Research Service, Grand Forks Human Nutrition Research Center Animal Care and Use Committee.

## Author Contributions

KC-L, JR, and BS designed the animal study. MB developed and performed the lipidomic analysis. The paper was prepared by MB and KC-L, with contributions by BS and JR. All authors have read and approved the manuscript.

## Funding

This work was funded by grant support from the USDA Agricultural Research Service Project #3062-51000-054-00D.

## Conflict of Interest

The authors declare that the research was conducted in the absence of any commercial or financial relationships that could be construed as a potential conflict of interest.

## Publisher’s Note

All claims expressed in this article are solely those of the authors and do not necessarily represent those of their affiliated organizations, or those of the publisher, the editors and the reviewers. Any product that may be evaluated in this article, or claim that may be made by its manufacturer, is not guaranteed or endorsed by the publisher.

## References

[1] Ornellas F Souza-Mello V Mandarim-de-Lacerda CA Aguila MB. Programming of Obesity and Comorbidities in the Progeny: Lessons From a Model of Diet-Induced Obese Parents. PloS One (2015) 10(4):e0124737. doi: 10.1371/journal.pone.0124737 25880318 PMC4399989

[2] Franco JG Fernandes TP Rocha CP Calvino C Pazos-Moura CC Lisboa PC. Maternal High-Fat Diet Induces Obesity and Adrenal and Thyroid Dysfunction in Male Rat Offspring at Weaning. J Physiol (2012) 590(21):5503–18. doi: 10.1113/jphysiol.2012.240655 PMC3515834 22869015

[3] Butruille L Marousez L Pourpe C Oger F Lecoutre S Catheline D. Maternal High-Fat Diet During Suckling Programs Visceral Adiposity and Epigenetic Regulation of Adipose Tissue Stearoyl-CoA Desaturase-1 in Offspring. Int J Obes (Lond) (2019) 43(12):2381–93. doi: 10.1038/s41366-018-0310-z 30622312

[4] Claycombe-Larson KJ Bundy AN Roemmich JN. Paternal High-Fat Diet and Exercise Regulate Sperm miRNA and Histone Methylation to Modify Placental Inflammation, Nutrient Transporter mRNA Expression and Fetal Weight in a Sex-Dependent Manner. J Nutr Biochem (2020) 81:108373. doi: 10.1016/j.jnutbio.2020.108373 32422425

[5] Claycombe-Larson KJ Bundy A Lance EB Darland DC Casperson SL Roemmich JN. Postnatal Exercise Protects Offspring From High-Fat Diet-Induced Reductions in Subcutaneous Adipocyte Beiging in C57Bl6/J Mice. J Nutr Biochem (2022) 99:108853. doi: 10.1016/j.jnutbio.2021.108853 34517093 PMC9040048

[6] Krout D Roemmich JN Bundy A Garcia RA Yan L Claycombe-Larson KJ. Paternal Exercise Protects Mouse Offspring From High-Fat-Diet-Induced Type 2 Diabetes Risk by Increasing Skeletal Muscle Insulin Signaling. J Nutr Biochem (2018) 57:35–44. doi: 10.1016/j.jnutbio.2018.03.013 29669306

[7] Howell KR Powell TL. Effects of Maternal Obesity on Placental Function and Fetal Development. Reproduction (2017) 153(3):R97–R108. doi: 10.1530/REP-16-0495 27864335 PMC5432127

[8] Mitanchez D Chavatte-Palmer P. Review Shows That Maternal Obesity Induces Serious Adverse Neonatal Effects and Is Associated With Childhood Obesity in Their Offspring. Acta Paediatrica (2018) 107(7):1156–65. doi: 10.1111/apa.14269 29421859

[9] Kelly Amy C Powell Theresa L Jansson T. Placental Function in Maternal Obesity. Clin Sci (2020) 134(8):961–84. doi: 10.1042/CS20190266 PMC8820171 32313958

[10] Calabuig-Navarro V Haghiac M Minium J Glazebrook P Ranasinghe GC Hoppel C. Effect of Maternal Obesity on Placental Lipid Metabolism. Endocrinology (2017) 158(8):2543–55. doi: 10.1210/en.2017-00152 PMC5551552 28541534

[11] Schaar A Sun Y Sukumaran P Rosenberger TA Krout D Roemmich JN. Ca2+ Entry *via* TRPC1 Is Essential for Cellular Differentiation and Modulates Secretion via the SNARE Complex. J Cell Sci (2019) 132(13):jcs231878. doi: 10.1242/jcs.231878 31182642 PMC6633397

[12] Nilius B Owsianik G. The Transient Receptor Potential Family of Ion Channels. Genome Biol (2011) 12(3):218. doi: 10.1186/gb-2011-12-3-218 21401968 PMC3129667

[13] Haimovitz-Friedman A Kolesnick RN Fuks Z. Ceramide Signaling in Apoptosis. Br Med Bull (1997) 53(3):539–53. doi: 10.1093/oxfordjournals.bmb.a011629 9374036

[14] Guenther GG Peralta ER Rosales KR Wong SY Siskind LJ Edinger AL. Ceramide Starves Cells to Death by Downregulating Nutrient Transporter Proteins. Proc Natl Acad Sci (2008) 105(45):17402–7. doi: 10.1073/pnas.0802781105 PMC2582319 18981422

[15] Krout D Schaar A Sun Y Sukumaran P Roemmich JN Singh BB. The TRPC1 Ca2+-Permeable Channel Inhibits Exercise-Induced Protection Against High-Fat Diet-Induced Obesity and Type II Diabetes. J Biol Chem (2017) 292(50):20799–807. doi: 10.1074/jbc.M117.809954 PMC5733613 29074621

[16] Holland WL Bikman BT Wang L-P Yuguang G Sargent KM Bulchand S. Lipid-Induced Insulin Resistance Mediated by the Proinflammatory Receptor TLR4 Requires Saturated Fatty Acid–Induced Ceramide Biosynthesis in Mice. J Clin Invest (2011) 121(5):1858–70. doi: 10.1172/JCI43378 PMC3083776 21490391

[17] Liebisch G Binder M Schifferer R Langmann T Schulz B Schmitz G. High Throughput Quantification of Cholesterol and Cholesteryl Ester by Electrospray Ionization Tandem Mass Spectrometry (ESI-MS/MS). Biochim Biophys Acta (2006) 1761(1):121–8. doi: 10.1016/j.bbalip.2005.12.007 16458590

[18] Sundaram S Zacek P Bukowski MR Mehus AA Yan L Picklo MJ. Lipidomic Impacts of an Obesogenic Diet Upon Lewis Lung Carcinoma in Mice. Front Oncol (2018) 8:134. doi: 10.3389/fonc.2018.00134 29868466 PMC5958182

[19] Bukowski MR Picklo MJ. Simple, Rapid Lipidomic Analysis of Triacylglycerols in Bovine Milk by Infusion-Electrospray Mass Spectrometry. Lipids (2021) 56(2):243–55. doi: 10.1002/lipd.12292 33169389

[20] Zacek P Bukowski M Johnson L Raatz SK Picklo M. Selective Enrichment of N-3 Fatty Acids in Human Plasma Lipid Motifs Following Intake of Marine Fish. J Nutr Biochem (2018) 54:57–65. doi: 10.1016/j.jnutbio.2017.11.002 29257986

[21] Picklo M Vallee Marcotte B Bukowski M de Toro-Martin J Rust BM Guenard F. Identification of Phenotypic Lipidomic Signatures in Response to Long Chain N-3 Polyunsaturated Fatty Acid Supplementation in Humans. J Am Heart Assoc (2021) 10(3):e018126. doi: 10.1161/JAHA.120.018126 33461307 PMC7955441

[22] Picklo MJ Hanson BK Bukowski MR. Simplified Mass Spectrometric Analysis of Ceramides Using a Common Collision Energy. Lipids (2019) 54(8):471–7. doi: 10.1002/lipd.12179 31342535

[23] Zacek P Bukowski M Mehus A Johnson L Zeng H Raatz S. Dietary Saturated Fatty Acid Type Impacts Obesity-Induced Metabolic Dysfunction and Plasma Lipidomic Signatures in Mice. J Nutr Biochem (2019) 64:32–44. doi: 10.1016/j.jnutbio.2018.10.005 30428423

[24] Lintonen TP Baker PR Suoniemi M Ubhi BK Koistinen KM Duchoslav E. Differential Mobility Spectrometry-Driven Shotgun Lipidomics. Anal Chem (2014) 86(19):9662–9. doi: 10.1021/ac5021744 25160652

[25] Zacek P Bukowski M Rosenberger TA Picklo M. Quantitation of Isobaric Phosphatidylcholine Species in Human Plasma Using a Hybrid Quadrupole Linear Ion-Trap Mass Spectrometer. J Lipid Res (2016) 57(12):2225–34. doi: 10.1194/jlr.D070656 PMC5321225 27688258

[26] Pang Z Chong J Zhou G David A Chang L Barrette M. MetaboAnalyst 5.0: Narrowing the Gap Between Raw Spectra and Functional Insights. Nucleic Acids Res (2021) 49(W1):W388–96. doi: 10.1093/nar/gkab382 PMC8265181 34019663

[27] Simmons DG. Postimplatnation Development of the Chorioallantoic Placenta. In: Croy BA Yamada AT DeMayo FJ Adamson SL, editors. The Guide to Investigation of Mouse Pregnancy, vol. p . New York: Academic Press (2014). p. 143–61.

[28] Elmore SA Cochran RZ Bolon B Lubeck B Mahler B Sabio D. Histology Atlas of the Developing Mouse Placenta. Toxicol Pathol (2022) 50(1):60–117. doi: 10.1177/01926233211042270 34872401 PMC8678285

[29] Munilla MA Herrera E. A Cholesterol-Rich Diet Causes a Greater Hypercholesterolemic Response in Pregnant Than in Nonpregnant Rats and Does Not Modify Fetal Lipoprotein Profile. J Nutr (1997) 127(11):2239–45. doi: 10.1093/jn/127.11.2239 9349853

[30] McMurry MP Connor WE Goplerud CP. The Effects of Dietary Cholesterol Upon the Hypercholesterolemia of Pregnancy. Metabolism (1981) 30(9):869–79. doi: 10.1016/0026-0495(81)90065-2 7266377

[31] Chapman D. Phase Transitions and Fluidity Characteristics of Lipids and Cell Membranes. Q Rev Biophys (1975) 8(2):185–235. doi: 10.1017/S0033583500001797 1103214

[32] Lee JJ Lambert JE Hovhannisyan Y Ramos-Roman MA Trombold JR Wagner DA. Palmitoleic Acid Is Elevated in Fatty Liver Disease and Reflects Hepatic Lipogenesis. Am J Clin Nutr (2015) 101(1):34–43. doi: 10.3945/ajcn.114.092262 25527748 PMC4266891

[33] Duarte JA Carvalho F Pearson M Horton JD Browning JD Jones JG. A High-Fat Diet Suppresses *De Novo* Lipogenesis and Desaturation But Not Elongation and Triglyceride Synthesis in Mice. J Lipid Res (2014) 55(12):2541–53. doi: 10.1194/jlr.M052308 PMC4242447 25271296

[34] Delgado TC Pinheiro D Caldeira M Castro MMCA Geraldes CFGC López-Larrubia P. Sources of Hepatic Triglyceride Accumulation During High-Fat Feeding in the Healthy Rat. NMR Biomed (2009) 22(3):310–7. doi: 10.1002/nbm.1327 19012281

[35] Chakraborty M Jiang X-C. Sphingomyelin and its Role in Cellular Signaling. In: D. C, editor. Lipid-Mediated Protein Signaling Advances in Experimental Medicine and Biology, vol. 991 . Dordrecht: Springer (2013). 10.1007/978-94-007-6331-9_1 23775687

[36] Sághy É Szőke É Payrits M Helyes Z Börzsei R Erostyák J. Evidence for the Role of Lipid Rafts and Sphingomyelin in Ca^2+^-Gating of Transient Receptor Potential Channels in Trigeminal Sensory Neurons and Peripheral Nerve Terminals. Pharmacol Res (2015) 100:101–16. doi: 10.1016/j.phrs.2015.07.028 26238178

[37] Del Gaudio I Sasset L Di Lorenzo A Wadsack C. Sphingolipid Signature of Human Feto-Placental Vasculature in Preeclampsia. Int J Mol Sci (2020) 21(3):1019. doi: 10.3390/ijms21031019 PMC7037072 32033121

[38] Lee JY Sohn KH Rhee SH Hwang D. Saturated Fatty Acids, But Not Unsaturated Fatty Acids, Induce the Expression of Cyclooxygenase-2 Mediated Through Toll-Like Receptor 4*. J Biol Chem (2001) 276(20):16683–9. doi: 10.1074/jbc.M011695200 11278967

[39] Weatherill AR Lee JY Zhao L Lemay DG Youn HS Hwang DH. Saturated and Polyunsaturated Fatty Acids Reciprocally Modulate Dendritic Cell Functions Mediated Through TLR4. J Immunol (2005) 174(9):5390. doi: 10.4049/jimmunol.174.9.5390 15843537

[40] Ajuwon KM Spurlock ME. Palmitate Activates the NF-κb Transcription Factor and Induces IL-6 and Tnfα Expression in 3T3-L1 Adipocytes. J Nutr (2005) 135(8):1841–6. doi: 10.1093/jn/135.8.1841 16046706

[41] Bikman BT Summers SA. Ceramides as Modulators of Cellular and Whole-Body Metabolism. J Clin Invest (2011) 121(11):4222–30. doi: 10.1172/JCI57144 PMC3204836 22045572

[42] Clarson LH Roberts VH Hamark B Elliott AC Powell T. Store-Operated Ca2+ Entry in First Trimester and Term Human Placenta. J Physiol (2003) 550(Pt 2):515–28. doi: 10.1113/jphysiol.2003.044149 PMC2343039 12766233

[43] Kawarabayashi Y Hai L Honda A Horiuchi S Tsujioka H Ichikawa J. Critical Role of TRPC1-Mediated Ca^2+^ Entry in Decidualization of Human Endometrial Stromal Cells. Mol Endocrinol (2012) 26(5):846–58. doi: 10.1210/me.2011-1259 PMC5417103 22474110

[44] Wolfrum C Kiehlmann E Pelczar P. TRPC1 Regulates Brown Adipose Tissue Activity in a Pparγ-Dependent Manner. Am J Physiol Endocrinol Metab (2018) 315(5):E825–E32. doi: 10.1152/ajpendo.00170.2017 29989850

[45] Brown SH Eather SR Freeman DJ Meyer BJ Mitchell TW. A Lipidomic Analysis of Placenta in Preeclampsia: Evidence for Lipid Storage. PloS One (2016) 11(9):e0163972. doi: 10.1371/journal.pone.0163972 27685997 PMC5042456

[46] Mehus AA Rust B Idso JP Hanson B Zeng H Yan L. Time-Restricted Feeding Mice a High-Fat Diet Induces a Unique Lipidomic Profile. J Nutr Biochem (2021) 88:108531. doi: 10.1016/j.jnutbio.2020.108531 33098972

[47] Vomhof-Dekrey E Darland D Ghribi O Bundy A Roemmich J Claycombe K. Maternal Low Protein Diet Leads to Placental Angiogenic Compensation *via* Dysregulated M1/M2 Macrophages and Tnfα Expression in Sprague-Dawley Rats. J Reprod Immunol (2016) 118:9–17. doi: 10.1016/j.jri.2016.08.009 27596280

