# Corrigendum: Lipidomic Analysis of TRPC1 Ca^2+^-Permeable Channel-Knock Out Mouse Demonstrates a Vital Role in Placental Tissue Sphingolipid and Triacylglycerol Homeostasis Under Maternal High-Fat Diet

**DOI:** 10.3389/fendo.2022.949419

**Published:** 2022-06-28

**Authors:** Michael R. Bukowski, Brij B. Singh, James N. Roemmich, Kate J. Claycombe-Larson

**Affiliations:** ^1^ USDA-ARS Grand Forks Human Nutrition Research Center, Grand Forks, ND, United States; ^2^ School of Dentistry, UT Health Science Center San Antonio, San Antonio, TX, United States

**Keywords:** infusion lipidomics, placental lipidome, TRPC1, sphingolipid metabolism, triacylglycerol

The original article contained an error. In the first sentence of the section on *Animal Protocol Design and Approval*, the control mouse strain was incorrectly identified as C57BL/6; the actual mouse strain used was B6129SF2/J. The sentence “Two-month-old female C57BL/6 mice (Envigo, Indianapolis, IN) were fed diets containing either 16% (normal-fat, NF) or 45% fat (high-fat, HF) for 12 weeks (**Table S1**).” should have read “Two-month-old female B6129SF2/J mice (Envigo, Indianapolis, IN) were fed diets containing either 16% (normal-fat, NF) or 45% fat (high-fat, HF) for 12 weeks (**Table S1**).”

The authors apologize for this error and state that this does not change the scientific conclusions of the article in any way. The original article has been updated.

## Publisher’s Note

All claims expressed in this article are solely those of the authors and do not necessarily represent those of their affiliated organizations, or those of the publisher, the editors and the reviewers. Any product that may be evaluated in this article, or claim that may be made by its manufacturer, is not guaranteed or endorsed by the publisher.

