# Supplementary material for: Lipidomic Analysis of TRPC1 Ca2+-Permeable Channel-Knock Out Mouse Demonstrates a Vital Role in Placental Tissue Sphingolipid and Triacylglycerol Homeostasis Under Maternal High-Fat Diet
Source: Front Endocrinol (Lausanne). 2022 Mar 10;13:854269. doi: 10.3389/fendo.2022.854269 (PMC8960927; doi:10.3389/fendo.2022.854269)
Supplement: Supplementary file 1 [file DataSheet_1.docx]

**Supplemental Information**

Lipidomic analysis of TRPC1 Ca^2+^ permeable channel-knock out mouse demonstrates a vital role in placental tissue sphingolipid and triacylglycerol homeostasis under maternal high-fat diet

Michael R. Bukowski^1 *^, Brij B. Singh^2^, James N. Roemmich^1^, Kate J. Claycombe-Larson^1*^

^1^USDA-ARS Grand Forks Human Nutrition Research Center, Grand Forks, ND, USA

^2^ School of Dentistry, UT Health Science Center San Antonio, San Antonio, TX 78257, USA

*Co-Correspondence: Michael R. Bukowski, Ph.D.

USDA-ARS Beltsville Human Nutrition Research Center

10300 Baltimore Avenue

RM. 117, BLDG. 307C, BARC-EAST

Beltsville, MD 20705

Email: [michael.bukowski@usda.gov](mailto:michael.bukowski@usda.gov)

Kate J. Claycombe-Larson

USDA-ARS, Grand Forks Human Nutrition Research Center Grand Forks, ND USA 58203 Tel.: 701-795-8298 Fax: 701-795-8395 Email: kate.larson@usda.gov

Keywords: Infusion Lipidomics, Placental Lipidome, TRPC1, Sphingolipid Metabolism, Triacylglycerol

**Materials**

HPLC-grade isopropanol, chloroform, butylated hydroxytoluene, and hexane were ordered from Sigma Aldrich (St. Louis, MO, USA) and used as received. HPLC-grade methanol was purchased from Honeywell (Muskegon, MI, USA). Silicic acid (200-325 mesh) was ordered from Clarkson Chromatography Products Inc. (South Williamsport, PA, USA). Internal standards for TAG and CE analysis, triheptadecanoin (TAG 51:0) and cholesteryl heptadecanoate (CE 17:0), respectively, were ordered from NuChek Prep Inc. (Elysian, MN, USA). Cholesterol-d7 (d7-Chol), N-heptadecanoyl-D-erythro-sphingosylphosphorylcholine (SM d18:1/17:0), N-heptadecanoyl-D-erythro-sphingosine (Cer d18:1/17:0), and the LIPIDMAPS standards were ordered from Avanti Polar Lipids (Alabaster, AL, USA).

**Sample preparation**

Sample preparation was performed with a hybrid automated workflow which employed two Gerstel MultiPurpose Sampler robotic workstations (Gerstel Inc., Linthicum Heights, MD, USA), herein labeled MPS1 and MPS2. MPS1 was equipped with 100 µL, 1000 µL, and 2500 µL syringes, a Peltier-cooled three drawer stack held at 4 °C, six 120 mL solvent reservoirs, and a QuickMix™ high-speed vortex mixer (Gerstel Inc.) MPS2 was identical to MSP1 except for the replacement of the mixer with a M-Vap™ solvent evaporation station held at 45 °C, and a gripping tool. Samples were weighed into 4 mL round bottom vials (Gerstel Inc., Linthicum Heights, MD, USA) and capped with a 13 mm polypropylene cap with a Teflon-backed silicone septa and magnetic ring to allow for automated transport between modules.

Frozen placental samples were thawed on water ice then weighed. Sample masses ranged from 3.69 mg to 27.23 mg and were used as received. Using MPS1 500 µL of aqueous homogenization buffer was added (0.9% KCl, 550 µmol/L diethylene triamine pentaacetic acid). Tissue was lysed manually using a probe sonicator (Fisher Scientific, Pittsburgh, PA, USA) in 1-second pulses. MPS1 delivered 100 µL of neutral lipid internal standard solution (4.81 nmol/sample CE 17:0, 2.79 nmol/sample TAG 51:0, and 6.98 nmol/sample d^7^-Chol) and 2.5 mL of 3:2 hexane:isopropanol (50 µmol/L BHT) to each homogenized sample. After vigorous mixing samples settled for 10 minutes to allow for phase separation. A 1.5 mL portion of the upper organic phase was transferred to a clean vial, then the extraction of the aqueous layer was repeated with an additional 2.5 mL of extraction solvent, and 1.5 mL of the second extract was combined with the first. Extracts were transferred to MPS2 where solvent was removed using the mVAP module (45 °C, 4 mbar, 25 min) and replaced with 1 mL chloroform. This stock solution was used for all subsequent sample preparation and a 500 µL portion of each sample was removed and stored 1.5-mL amber glass vial for polar lipid analysis. The remaining stock received 84 mg of silicic acid using a Redding 10X pistol & small rifle powder measure (Cortland, NY, USA), and sample was returned to MPS1, which delivered 1 mL of additional chloroform and vortex mixed samples for 1 minute followed by a settling time of 10 minutes. After the dispersive SPE matrix settled 100 µL of the supernatant was combined with 100 µL of methanol (20 mM ammonium acetate) in amber glass autosampler vial. This sample (Solution 1) was analyzed for triacylglycerol and cholesterol ester content as described below.

The assay for unesterified cholesterol was performed using a modification of the method published by Liebisch *et al.[1]*. A 500 µL portion of the SPE supernatant was transferred to a clean tube and combined with 200 µL of 1:5 acetyl chloride: chloroform (v/v) and incubated at room temperature for two hours. Solvent was removed under a gentle flow of argon in a 30 °C water bath using an N-Evap evaporator (Organomation, Berlin, MA, USA) in a fume hood. Dried samples were re-dissolved in 250 µL of 1:1 chloroform: methanol (Solution 2). This portion of the experiment was performed manually due to the strongly corrosive nature of the acetyl chloride solution.

Analysis of polar lipids was performed using the method adapted from Sundaram *et al.* with modifications made to allow for automation. [2] Using MPS1, 50 µL of stock extract were transferred to an autosampler vial pre-filled with 940 µL of 1:1 chloroform: methanol (10 mM ammonium acetate). To this solution (Solution 3) 10 µL of chloroform internal standard solution containing Cer d18:1/17:0 (34.1 nmol/sample) and SM d18:1/17:0 (78.1 pmol/sample) were added. To avoid interference from the SM internal standard during analysis of the PC species, a sample for PC and PE analysis was generated through serial dilution. A 50 µL of Solution 4 received 10 µL of internal standard solution containing PE 17:0/14:1 (14.6 pmol/sample), PE 17:0/20:4 (13.2 pmol/sample), PC 17:0/14:1 (28.9 pmol/sample), and PC 17:0/20:4 (24.9 pmol/sample).

**Instrumentation**

Data were collected on an AB Sciex 5500 QTRAP hybrid mass spectrometer equipped with a Turbo V electrospray ion source and SelexION ion mobility device (AB Sciex, Framingham, MA, USA). Samples were infused using a Shimadzu Prominence UPLC system (Shimadzu Scientific Instruments, Columbia, MD, USA) equipped with an LC20XR autosampler (50 µL stainless steel sample loop) and two solvent delivery units following a configuration modified from Bukowski and Picklo [3]. Briefly, pump A drove an admixture of 1:1 methanol chloroform through the autosampler at a rate of 20 µL per minute. Pump B drove a solution of 1:1 methanol: chloroform containing 10 mM ammonium acetate at a rate of 50 µL/min, joining the autosampler output at a T-junction prior to the electrospray source. All connections were made using 100 µm PeekSil tubing. Due to the high chloroform content, degassing units were bypassed. The ESI source operated in positive mode at 5500 V, 250 °C, with gas flows of 25, 15, and 20 units for curtain gas, GS1, and GS2, respectively. The Selexion device was employed for the analysis of sphingomyelin species as detailed below.

Multiple instrument modes were used based upon lipid class and preparation (Table S2). Triacylglycerols (TAG) were characterized and quantitated by brutto structure as ammoniated cations, [TAG + NH4]^+^ in enhanced mass spectrum (EMS) mode as previously published [3, 4]. Cholesterol esters (CE) were assayed by neutral loss scan for 20 fatty acid neutral losses with confirmatory detection of the cholesterol head group by product ion scan for m/z = 369, as previously published (Table S3)[4, 5]. Acylated samples for unesterified cholesterol (FC) determination were analyzed using multiple reaction monitoring for the acylated d7-cholesterol species (*m/z* = 453.4🡪376.3) and the endogenous acylated cholesterol (*m/z* = 446.4🡪369.3)[1]. Ceramide species (Cer) were detected using the product ion m/z 264, selective to the sphingosine backbone, and quantified was performed following the method of Picklo et al.[6]. Phosphatidylethanolamine, and phosphatidylcholine species were quantified as brutto species using our previously published methods [4, 7]. Monitoring the neutral loss of 141 Da allowed for the selective measurement of PE species, while monitoring the product ion m/z = 184 was selective for phosphatidylcholine and sphingomyelin species.

Sphingomyelin species were isolated for characterization and quantitation using the Selexion ion mobility device [8]. In addition to the source parameters above, the separation voltage was set to 3900 V and a compensation voltage of 6.8 V was applied. A modifier of 1-propanol was used (molecular weight 60.1, density = 0.804) at the “low” setting, the DMS cell temperature and resolution gas were both operated at the “low” setting. Response factors were determined using commercially available SM standards where available; for SM species with no commercially available equivalent, response factors were assigned based upon the nearest commercial standard by mass.

**Data processing**

Mass spectra for all lipid class were examined manually to confirm brutto structure assignment. Briefly, for each class-selective scan spectra were summed for all samples in Peak View visualization software (AB Sciex, Framingham, MA, USA). This composite spectrum was used to generate a consensus target list of brutto structures for each lipid class. The naming Isotopic and ionization correction factors were determined as previously described and quantitation of target species was performed using LipidView software (AB Sciex, Framingham, MA, USA)[3, 4, 6, 9]. Values were normalized to tissue wet weight. TAG 54:0 and TAG 54:1 were excluded from analysis due to isobaric overlap with a silicone oligomer contaminant which was extracted from the septa and could not be reliably removed by subtraction.

**Identification of PC and PE species**

For the PC spectrum signal intensity was distributed among species with 34, and 36 acyl carbons with between 0 and 3 double bonds, corresponding to structures containing 16- and 18-carbon fatty acids such as palmitic, palmitoleic, stearic, oleic, and linoleic acids. An additional cluster of peaks representing 38 acyl carbon species was dominated by PC 38:4, a species identified as PC 18:0_20:4 based upon identification of the anions [FA 20:4]-, [FA 20:4 -CO_2_]-, and related characteristic fragments in negative ESI MS^3^ scans (Table S9) [9]. The complementary PE 18:0_20:4 species was identified in the PE spectrum using a negative ESI MS^2^ scan [9]. The dominant features in the PE spectrum were species with 40 acyl carbons and between 4 to 7 double bonds, which suggested a great degree of long chain PUFA incorporation into PE. Ions characteristic of [FA 22:X]^-^, [FA 22:X -CO_2_]^-^ where X = 4, 5, or 6 double bonds were detected using negative ESI MS^2^ for PE 40:5, PE 40:6, and PE 40:7, respectively, confirming the presence of long-chain PUFA fatty acids in these PE species.

**References**

1. Liebisch, G., et al., *High throughput quantification of cholesterol and cholesteryl ester by electrospray ionization tandem mass spectrometry (ESI-MS/MS).* Biochim Biophys Acta, 2006. **1761**(1): p. 121-8.

2. Sundaram, S., et al., *Lipidomic Impacts of an Obesogenic Diet Upon Lewis Lung Carcinoma in Mice.* Front Oncol, 2018. **8**: p. 134.

3. Bukowski, M.R. and M.J. Picklo, *Simple, Rapid Lipidomic Analysis of Triacylglycerols in Bovine Milk by Infusion-Electrospray Mass Spectrometry.* Lipids, 2021. **56**(2): p. 243-255.

4. Zacek, P., et al., *Selective enrichment of n-3 fatty acids in human plasma lipid motifs following intake of marine fish.* J Nutr Biochem, 2018. **54**: p. 57-65.

5. Picklo, M., et al., *Identification of Phenotypic Lipidomic Signatures in Response to Long Chain n-3 Polyunsaturated Fatty Acid Supplementation in Humans.* J Am Heart Assoc, 2021. **10**(3): p. e018126.

6. Picklo, M.J., B.K. Hanson, and M.R. Bukowski, *Simplified Mass Spectrometric Analysis of Ceramides using a Common Collision Energy.* Lipids, 2019. **54**(8): p. 471-477.

7. Zacek, P., et al., *Dietary saturated fatty acid type impacts obesity-induced metabolic dysfunction and plasma lipidomic signatures in mice.* J Nutr Biochem, 2019. **64**: p. 32-44.

8. Lintonen, T.P., et al., *Differential mobility spectrometry-driven shotgun lipidomics.* Anal Chem, 2014. **86**(19): p. 9662-9.

9. Zacek, P., et al., *Quantitation of isobaric phosphatidylcholine species in human plasma using a hybrid quadrupole linear ion-trap mass spectrometer.* J Lipid Res, 2016. **57**(12): p. 2225-2234.

**Figure S1. Placental phosphatidylcholine concentration by species for PC C:N.** C = acyl carbon number and N = acyl desaturation level. Data are shown as mean ± sem (n = 16, except for HF-TRPC1 where n = 12). Symbols indicate differences with p<0.05 base upon one-way ANOVA with Tukey’s HRD post hoc test and application of a 0.05 false discovery rate for gestational age (*), diet (**), and genotype (‡). P-values are available in supplemental materials.

**Figure S2. Placental phosphatidylethanolamine concentration by species for PE C:N.** C = acyl carbon number and N = acyl desaturation level. Data are shown as mean ± sem (n = 16, except for HF-TRPC1 where n = 12). Symbols indicate differences with p<0.05 base upon one-way ANOVA with Tukey’s HRD post hoc test and application of a 0.05 false discovery rate for gestational age (*), diet (**), and genotype (‡). P-values are available in supplemental materials.

**Figure S3. Additional Placental triacylglycerol concentration by species for TAG C:N.** C = acyl carbon number and N = acyl desaturation level. Data are shown as mean ± sem (n = 16, except for HF-TRPC1 where n = 12). Symbols indicate differences with p<0.05 base upon one-way ANOVA with Tukey’s HRD post hoc test and application of a 0.05 false discovery rate for gestational age (*), diet (**), and genotype (‡). P-values are available in supplemental materials.
